# Role of StAR Gene in Sex Steroid Hormone Regulation and Gonadal Development in Ark Shell *Scapharca broughtonii*

**DOI:** 10.3390/biology14080925

**Published:** 2025-07-23

**Authors:** Wenjing Wang, Zhihong Liu, Huaying Zhang, Zheying Gao, Sudong Xia, Xiujun Sun, Liqing Zhou, Zhuanzhuan Li, Peizhen Ma, Biao Wu

**Affiliations:** 1 State Key Laboratory of Mariculture Biobreeding and Sustainable Goods, Yellow Sea Fisheries Research Institute, Chinese Academy of Fishery Sciences, Qingdao 266071, China; 17806253665@163.com (W.W.); liuzh@ysfri.ac.cn (Z.L.); xjsun@ysfri.ac.cn (X.S.); zhoulq@ysfri.ac.cn (L.Z.); lizz@ysfri.ac.cn (Z.L.); mapz@ysfri.ac.cn (P.M.); 2 Laboratory for Marine Fisheries Science and Food Production Processes, Qingdao National Laboratory for Marine Science and Technology, Qingdao 266237, China; 3 Tianjin Agricultural Development Service Center, Tianjin 300061, China; zhanghuaying2007@126.com; 4 Tianjin Binhai New Area Agricultural and Rural Development Service Center, Tianjin 300452, China; xqnwsc@163.com; 5 Tianjin Key Lab of Aqua-Ecology and Aquaculture, Department of Fishery Science, Tianjin Agricultural University, Tianjin 300384, China; xsd20022003@126.com

**Keywords:** *Scapharca broughtonii*, *StAR*, sex steroid hormones, gonadal development

## Abstract

This study elucidates the role of the *steroidogenic acute regulatory protein* (*StAR*) in sex steroid hormone dynamics and the gonadal development of the commercially important marine bivalve ark shell *Scapharca broughtonii*. The sequence of the *StAR* gene was obtained and verified from the transcriptome of ark shell, then the tissue localization and expression pattern during the gonad development of the *StAR* gene were detected by in situ hybridization and quantitative real-time PCR, respectively. Additionally, the concentrations of three critical sex steroid hormones (progesterone, testosterone, and estradiol) were measured throughout gonadal development using enzyme-linked immunosorbent assay (ELISA). The results showed that the length of the coding region of *StAR* was 1446 bp, encoding 481 amino acids. The results of qRT-PCR showed that the expression of the *StAR* gene varied with gonadal development, increased from the early active stage to the development stage, and decreased from the mature stage to the spent stage. Notably, the expression level in ovaries was higher than that in testes, suggesting the potential involvement of *StAR* in sex differentiation and gonadal development. Additionally, the results indicated that progesterone, testosterone, and estradiol accounted for 80%, 10%, and 10% of the total hormone content in the gonads, respectively. Correlation analysis revealed a highly significant strong positive correlation between progesterone/estradiol levels and *StAR* gene expression, demonstrating that *StAR* serves as a key regulator in sex steroid hormone biosynthesis. These findings provide crucial molecular evidence for *StAR*-mediated steroidogenesis in bivalve reproduction, offering fundamental insights into invertebrate endocrinology.

## Simple Summary

Sex steroid hormones play pivotal roles in regulating reproductive cycles in vertebrates, yet their seasonal dynamics and functional significance in shellfish remain poorly characterized. The ark shell (*Scapharca broughtonii*) is one of the most economically important mollusks in the Bohai Sea and Yellow Sea of China. Studying the regularity and regulation of gonadal development in this economically important bivalve is crucial for artificial seedling breeding. This study elucidates the role of the *steroidogenic acute regulatory protein* (*StAR*) in sex steroid hormone dynamics and gonadal development in ark shell, while simultaneously quantifying three principal sex steroid hormones (progesterone, testosterone, and estradiol) throughout gonadal development. The results demonstrated that *StAR* potentially regulates gonadal maturation by modulating steroid hormone biosynthesis. These findings provide crucial molecular and novel insights into *StAR*-mediated steroidogenesis and endocrine mechanisms governing bivalve reproduction, with implications for aquaculture management and conservation.

## 1. Introduction

Gonadal development in aquatic animals is regulated by both genetic factors and exogenous environmental factors, including temperature, salinity, pH, and light cycle [1]. Sex steroid hormones are widely present in vertebrates and regulate the expression of downstream target genes via steroid receptor-mediated signaling pathways, thereby modulating various physiological processes, such as reproduction and development [2]. In recent years, there has been increasing research on genes related to sex steroid hormone synthesis in vertebrates, but relatively few studies have focused on shellfish [3]. Initially, many scholars believed that sex steroid hormones were exclusive to vertebrates; however, accumulating evidence suggests that they are also present in invertebrates [4,5]. Studies have reported the presence of sex steroid hormones in the gonads of Yesso scallop (*Mizuhopecten yessoensis*) [6], Zhikong scallop (*Chlamys farreri*) [7], Bay scallop (*Argopecten irradians*) [8], Pacific oyster (*Crassostrea gigas*) [9], and Jinjiang oyster (*Crassostrea ariakensis*) [10]. These findings indicate that the levels of sex steroid hormones in the gonads fluctuate seasonally in accordance with the reproductive cycle. The synthesis of sex steroid hormones uses cholesterol as a precursor and is catalyzed and regulated by a series of steroid synthesis enzymes, primarily belonging to the cytochrome P450 (CYP) gene families and hydroxysteroid dehydrogenase (HSD) gene families, along with other steroid oxidoreductases [11]. Steroid synthetase and its pathways have been extensively studied in vertebrates [12]. First, cholesterol is transported from cytoplasm to the mitochondrial inner membrane under the action of steroidogenic acute regulatory protein (*StAR*) for pregnenolone synthesis [13]. Subsequently, cholesterol is catalyzed by the Cholesterol Side-chain Cleavage Enzyme (*CYP11A*) to form pregnenolone. Once pregnenolone returns to the cytoplasm, the corresponding sex steroid hormones are synthesized through the action of steroid synthetases encoded by *CYP17*, *CYP19*, *3β-HSD,* and *17β-HSD* [14]. Studies have shown that prominent mollusk species, such as Cephalopods, Gastropods, and bivalves, possess the ability to synthesize sex steroid hormones using precursors derived from cholesterol or pregnenolone [15]. However, research on the synthetic molecular mechanism of sex steroid hormones in shellfish is relatively limited, and further investigation is required into the key genes involved in the synthetic pathway of sex steroid hormones.

The *StAR* gene encodes the steroidogenic acute regulatory protein, a transporter protein situated on the mitochondrial membrane. The transportation of cholesterol from the outer mitochondrial membrane to the inner membrane by *StAR* is the initial and rate-limiting step in the synthesis of sex steroid hormones [16]. In mammals, the regulation of the *StAR* gene is governed by both the hypothalamus–pituitary–adrenal (HPA) axis and the hypothalamic–pituitary–gonadal (HPG) axis [17]. Furthermore, the expression of the *StAR* gene is modulated by various factors, including the *SF-1* gene, which has been shown to regulate *StAR* gene expression [18]. In vertebrates such as zebrafish (*Danio rerio*), medaka (*Oryzias latipes*), and Nile tilapia (*Oreochromis niloticus*), there are typically two *StAR* genes, designated as *StAR1* and *StAR2*. *StAR1* is predominantly expressed in head kidney tissues and plays a role in cortisol production, while *StAR2* is primarily expressed in gonads and is crucial for hormone production during gonadal differentiation and development [19]. Current evidence suggests that the number of *StAR* subtypes in mollusks varies across species, and different subtypes may play distinct roles in processes such as gonadal development and stress response. In shellfish, *StAR3* genes have been reported to be related to steroid hormone synthesis. In *C. farreri*, the expression of the *StAR3* gene during ovarian differentiation and development was detected, revealing that its expression continuously changes with ovarian growth, potentially providing an essential enzyme for sex steroid hormone synthesis during early ovarian cell differentiation and ovarian maturation [20]. After interfering with the *StAR3* gene in *Hyriopsis cumingii*, it was observed that the *StAR3* gene influenced the expression of downstream genes, such as *CYP17A* and *17β-HSD11*, as well as the levels of sex steroid hormone contents in the gonads. This suggests that the *StAR3* gene may act upstream of *CYP17A* and *17β-HSD11* in the sex steroid hormone synthesis pathway and participate in this process, indicating the presence of a similar sex hormone synthesis pathway in *H. cumingii* as in vertebrates [21].

The ark shell, *Scapharca broughtonii*, is not only an important large-scale benthic shellfish in the Yellow and Bohai Sea areas of China but also a vital economic aquaculture species in Northern China. It has significant fishery value, nutritional value, and ecological functions. With its delicious meat, it is rich in protein and various minerals and is widely popular in domestic and international markets [22]. Due to its gonads being covered by the foot, they are not visible to the naked eye without dissection, which poses challenges for accurately assessing gonad development. This issue has become a significant obstacle in artificial seedling rearing. If we can establish an evaluation system based on molecular and hormonal levels for gonadal development in ark shell, implement the non-invasive diagnosis of sex and developmental stages to accurately determine gender and developmental phases, and use hormonal markers to identify the stages of gonadal development, it will further optimize seedling rearing management, improve breeding efficiency, and reduce production costs. This will provide important technical support for the artificial propagation of ark shell and address the technical bottleneck of traditional reliance on anatomical observation. Sex steroid hormones, as critical regulators of sexual maintenance and gonad development in aquatic organisms, play a pivotal role in the reproductive biology of shellfish. However, there is currently limited research on these aspects in ark shell. Investigating the correlation between gonadal development and sex steroid hormone-related genes in this species could provide valuable insights for the successful selection and breeding of artificial shellfish populations. In this study, we have screened and validated the sequence of the *StAR* gene in ark shell, and then analyzed its bioinformatics and the spatiotemporal expression pattern within the gonads using in situ hybridization and qRT-PCR techniques. Additionally, a method for the quantifying sex steroid hormones (progesterone, testosterone, and estradiol) in ark shell has been established to determine and analyze the variation in the content and proportion of these sex steroid hormones in the ovaries and testes during different gonad development stages. These findings provide a solid foundation for understanding the role of the *StAR* gene in gonad development in ark shell and contribute to elucidating the functions of sex steroid hormones and their associated genes in the reproductive processes of marine bivalves.

## 2. Materials and Methods

### 2.1. Samples

A total of 200 ark shells with an average shell length of 80.74 ± 5.08 mm were collected from the Qingdao sea area in Shandong Province, China, for experimental analysis. This study was conducted from March 2021 to September 2021, covering the complete reproductive cycle of ark shell in the Yellow Sea region. Prior to sampling, the ark shells were acclimated in tanks filled with aerated and filtered seawater under controlled conditions: salinity of 30 ± 1, temperature of 18 ± 1 °C, pH of 7.75 ± 0.25, and dissolved oxygen levels of 7.56 ± 0.23 mg/L. This acclimation period lasted one week. During this time, the seawater was fully refreshed daily with fresh aerated and filtered seawater, and the ark shells were fed single-celled algae twice daily. Following gonad tissue sampling, histological analysis divided the developmental stages of the gonad of ark shell into five distinct phases: early active stage (stage Ⅰ), development stage (stage Ⅱ), ripe stage (stage Ⅲ), spawning stage (stage Ⅳ), and spent stage (stage Ⅴ). Additionally, samples from seven tissues including testis (stage Ⅲ), ovary (stage Ⅲ), gill, foot, mantle, muscle, and hepatopancreas were collected and fixed in situ hybridization fixative (Servicebio, Wuhan, China) for 24 h to prepare for the in situ hybridization experiment.

### 2.2. RNA Extraction and cDNA Synthesis

RNA was extracted from three spermatic and ovarian samples at each of the five gonadal development stages in ark shell. The extraction was performed using the Trizol method, and RNA quality was assessed by 1% agarogel electrophoresis for integrity and Nanodrop 2000 (Thermo Scientific, Waltham, MA, USA) for purity and concentration. A satisfactory OD_260_/OD_280_ ratio should fall within the range of 1.8 to 2.2. A qualified sample was diluted to 1000 ng/μL and used as a template for reverse transcription reactions, which were carried out using the Evo M-MLV Plus cDNA synthesis kit (Accurate, Changsha, China). The cDNA was stored at −20 °C for subsequent analyses.

### 2.3. cDNA Fragment Validation

From the early transcriptome sequencing results (BioProject ID: PRJNA948553), cDNA fragments of the *StAR* gene were identified in the ark shell transcriptome library. Specific primers were designed using Primer 3.0 (http://www.primer3plus.com) URL (accessed on 10 July 2022). The primers used for cDNA fragment validation are listed in Table 1. The target gene was amplified using 2 × Tap Plus Master Mix Ⅱ (Dye Plus) (Vazyme, Nanjing, China), and the PCR products were analyzed by 1% agarose gel electrophoresis. Subsequently, the TaKaRa MiniBEST Agarose Gel DNA Extraction kit (TaKaRa, Beijing, China) was employed for gel extraction. The purified target fragment was ligated into a vector using a 5 min TA/Blunt Zero Cloning kit (Vazyme, Nanjing, China) and then transferred to DH5α competent cells (Vazyme, Nanjing, China). After overnight incubation on LB plates containing ampicillin (*Amp*), positive colonies were selected and sent to Beijing Tsingke Biotechnology Co., Ltd. (Beijing, China) for sequencing.

### 2.4. Gene Sequence and Evolutionary Analysis

According to the *StAR* gene sequence obtained through sequencing analysis, the ORF Finder (https://www.ncbi.nlm.nih.gov/orffinder/orffinder/) URL (accessed on 18 July 2022) was employed for open reading frame (ORF) prediction. The SignaIP 5.0 Server (https://services.healthtech.dtu.dk/service.php?SignalP-5.0) URL (accessed on 15 September 2022) was used to predict the presence of signal peptides. TMHMM 2.0 (https://services.healthtech.dtu.dk/services/TMHMM-2.0/) URL (accessed on 15 September 2022) was applied for predicting the protein transmembrane domain structure. SMART (http://smart.emblheidelberg.de/smart/set_mode.cgi?NORMAL=1) URL (accessed on 15 September 2022) was used to analyze gene structure and functional domains. The ExPASY tool (https://web.expasy.org/protparam/) URL (accessed on 15 September 2022) was employed to predict the physical and chemical properties of the protein. The SWISS-MODEL (https://www.swissmodel.expasy.org/) URL (accessed on 15 September 2022) website was used for predicting protein tertiary structure. Subcellular localization was predicted using the Cell-PLoc 2.0 server (http://www.csbio.sjtu.edu.cn/bioinf/Cell-PLoc-2/) URL (accessed on 15 September 2022). BLASTp (https://blast.ncbi.nlm.nih.gov/Blast.cgi) URL (accessed on 15 September 2022) was utilized for nucleotide sequence homology analysis and the identification of similar protein sequences. The MEGA 11 was used for phylogenetic tree construction.

### 2.5. In Situ Hybridization Analysis

In situ hybridization was used to analyze the cytological location of the *StAR* gene in the testis, ovary, gill, foot, mantle, muscle, and hepatopancreas of ark shell. The hybridization probe was designed by Wuhan Servicebio Technology Co., Ltd. (Wuhan, China), and its sequence is presented in Table 1. The main experimental procedures were as follows: fixed tissues were dehydrated using a gradient alcohol series, embedded in paraffin after wax immersion, sectioned, baked, and then processed sequentially with xylene (15 min twice) and anhydrous ethanol (5 min, twice), followed by air-drying and soaking in DEPC-treated water. Tissue sections were boiled in a repair solution for 5 min and cooled naturally. Based on the specific characteristics of different tissues, samples were digested with protease K at 37 °C for 15 min. After rinsing with distilled water, the samples were washed three times with 1 × PBS and pre-hybridized with pre-hybridization solution at 37 °C for 1 h. Subsequently, the samples were incubated overnight at 42 °C in hybridization solution containing a 500 nM probe. Following the removal of the hybridization solution, standard probe incubation was performed with two standard probes (1:400 dilution) at 42 °C for 3 h. After blocking with normal rabbit serum, the samples were incubated with mouse anti-digoxin labeled alkaline phosphatase anti-DIG-AP (1:400 dilution) at 37 °C for 50 min. Color development was performed with a 2% NBT/BCIP solution in darkness at room temperature for 2 h. After rinsing with pure water and natural drying, the section was sealed with neutral gum. The sections obtained from in situ hybridization were observed under a CIC microscope (XSP-C204) for image acquisition and analysis.

### 2.6. Analysis of StAR Expression in the Gonads of Ark Shell Across Different Developmental Stages

Primers for qRT-PCR were designed based on the coding sequence (CDS) obtained from sequencing. The primers used for qRT-PCR are listed in Table 1, with the RL15 gene serving as the reference gene [23]. Total RNA was reverse-transcribed into cDNA using HiScript^®^ Ⅲ RT SuperMix for the qPCR kit (Vazyme, Nanjing, China). Subsequently, qRT-PCR was performed on CFX 96 (Bio-rad, Hercules, CA, USA) using ChamQ SYBR Color qPCR Master Mix kit (Vazyme, Nanjing China). The reaction system consisted of 10 μL of 2 × ChamQ SYBR Color qPCR Master Mix, 0.4 μL each of positive and negative primers, 1 μL of cDNA template, and 8.2 μL of RNase Free H_2_O. The thermal cycling conditions were as follows: initial denaturation at 95 °C for 3 min, followed by 40 cycles of 95 °C for 10 s and 60 °C for 30 s. Each qRT-PCR experiment was conducted in triplicate, and the relative expression level of the *StAR* gene was calculated using the 2^−∆∆Ct^ method.

### 2.7. Extraction of Sex Steroid Hormone

The sex steroid hormone was extracted using the dichloromethane liquid–liquid extraction technique. Three samples were randomly selected from each of the five gonadal developmental stages in both testes and ovaries. A total of 0.2 g of gonadal tissue was weighed and mixed with 500 μL of ultra-pure water, followed by homogenization using Tissuelyser (Jingxin, Shanghai, China) at a frequency of 70 Hz for 2 min (repeated four times). The homogenized sample was then disrupted using an ultrasonic cell disruptor. Subsequently, 400 μL of preheated HCl (30 °C) was added, mixed thoroughly for 2 min, and incubated in a water bath at 40 °C for 15 min. The homogenate was transferred to a 50 mL centrifuge tube, and 1.25 mL of 0.07 mol/L Na_2_HPO_4_ solution and 14 mL of dichloromethane solution were added. After swirling for 2 min, the mixture was centrifuged at 6000 rpm for 10 min. The lower organic phase was carefully transferred to another 50 mL centrifuge tube. An additional 14 mL of dichloromethane was added to the remaining upper inorganic phase, and the mixture was swirled for 2 min before being centrifuged again at 6000 rpm for 10 min. The lower organic phase was collected into the 50 mL centrifuge tube. The dichloromethane in the organic phase was evaporated under nitrogen, and the resulting solid residue was dissolved in 500 μL of ELISA buffer.

### 2.8. Determination of Sex Steroid Hormone

The concentrations of testosterone, progesterone, and estradiol in the gonads were measured using the Cayman enzyme-linked immunosorption assay (ELISA) kit (Cayman, Ann Arbor, MI, USA) according to the manufacturer’s instructions. Standard substances were subjected to gradient dilution, followed by the sequential addition of reagent, sample, tracer, and antiserum onto a plate coated with mouse anti-rabbit IgG. The plate was incubated at room temperature in a dark environment with oscillations for 1 h. Subsequently, all solutions were aspirated, and the plate was washed with Wash Buffer. Developer and tracer were then added, and the plate was incubated on an oscillator at room temperature for 10–15 min. Absorbance was measured at the corresponding wavelengths for each hormone using a MultiskanTM FC enzyme labeler (Thermo Scientific™, Waltham, MA, USA). Each sample was assayed in duplicate, and the hormone concentrations in gonad tissue were calculated using the ELISA double table provided by Cayman. Sex steroid hormone content was expressed as the amount of steroids per gram of gonad wet weight (pg/g) (the gonad tissue was weighed within 2 min after dissection).

### 2.9. Statistical Analysis

The significance of differences in sex steroid hormones across different gonadal development stages was assessed using one-way ANOVA. The significance of differences in sex steroid hormones content between ovary and testis at the same development stage was evaluated using an independent sample t-test with a significance level set at *p* < 0.05. Post hoc comparisons were conducted using Duncan multiple comparisons. Correlation analysis was employed to determine the relationship among the three hormone levels and gene expression levels. All statistical analysis was performed using SPSS 22.0 software. Quantitative data are presented as mean ± standard deviation (SD), and the threshold for statistical significance was established as *p* < 0.05.

## 3. Results

### 3.1. StAR Gene Sequence and Bioinformatics Analysis

**Figure 1:**
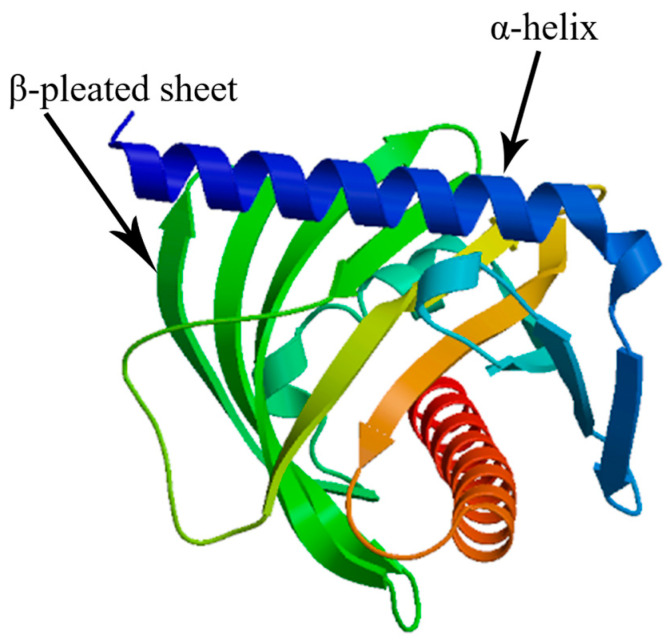
Prediction of tertiary structure of StAR protein.

The cloned CDS of the *StAR* gene was 1446 bp in length, encoding a protein consisting of 481 amino acids (Genbank accession number: PV867238). The physicochemical properties of the *StAR* protein were predicted using ExPASY online software, and the analysis revealed that the *StAR* protein lacks signal peptide but contains four transmembrane helices, with a total probability of 96.74% that the N-term is located on the cytoplasmic side of the membrane. The molecular weight of the *StAR* protein was calculated to be 55.24 kDa, with a theoretical isoelectric point of 6.04. Its molecular formula is C_2479_H_3788_N_666_O_722_S_24_, and it exhibits a lipolysis coefficient of 79.65, a grand average hydropathicity (GRAVY) value of −0.294, and an instability coefficient of 48.21. These characteristics indicate that the *StAR* protein is hydrophilic and unstable. Additionally, the *StAR* gene contains a conserved lipid transfer functional domain (SMART) within amino acid residues 270~477. Subcellular localization analysis demonstrated that the *StAR* protein resides in the cytoplasm, likely associated with its cholesterol transport function. Furthermore, SWISS-MODEL prediction results showed that the tertiary structure of the *StAR* protein consists of α-helix with multiple β-pleated sheet (Figure 1). The results of subcellular localization analysis indicated that the StAR protein is present in the cytoplasm, which may be related to its function in cholesterol transport.

**Figure 2:**
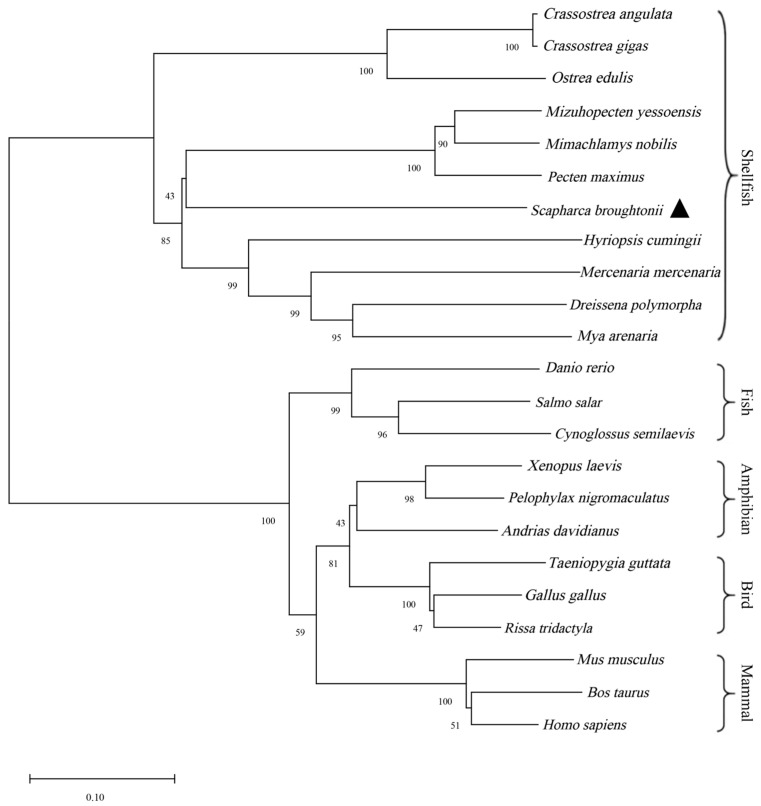
Phylogenetic tree of StAR amino acid sequence in different species.

A homology analysis of the amino acid sequence of the *StAR* protein conducted by BLAST demonstrated that the *StAR* gene of ark shell exhibited the highest sequence similarity (51.85%) with *Dreissena polymorpha*. Moderate homology levels were observed between the ark shell *StAR* gene and other molluscan species, including *M. yessoensis*, *Mimachlamys nobilis*, *Pecten maximus*, *H. cumingii*, *Mercenaria mercenaria Mya arenaria,* and oysters, with values ranging from 46.17% to 51.45% (Table 2). MEGA 11 software was adopted to construct the phylogenetic tree of the *StAR* gene of ark shell and other species by adopting the Neighbor-Joining (NJ) method (Figure 2). The results indicated that the *StAR* gene of ark shell was first clustered into a branch of shellfish such as scallops, oysters, and clams, then had a distant evolutionary relationship with fishes, amphibians, birds, and mammals.

### 3.2. Cytological Mapping of StAR Gene mRNA in Seven Tissues of Ark Shell

**Figure 3:**
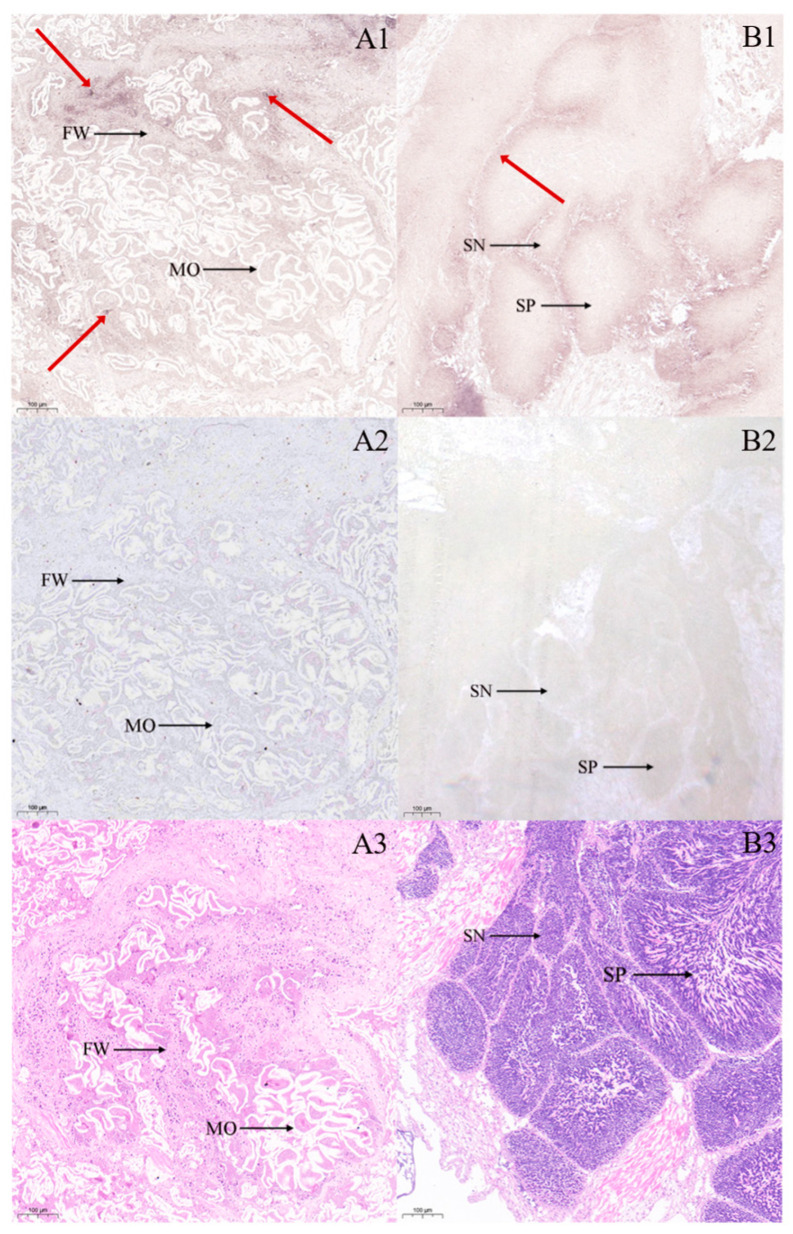
Cytological localization of *StAR* in gonad of ark shell. Note: Positive signals are indicated by the red arrows. (**A**): Ovary; (**B**): testis; (**1**): in situ hybridization results; (**2**): negative control diagram; (**3**): hematoxylin–eosin staining; MO: mature oocyte; FW: follicle wall; SN: spermatogonia; SP: sperm; bar: 100 µm.

**Figure 4:**
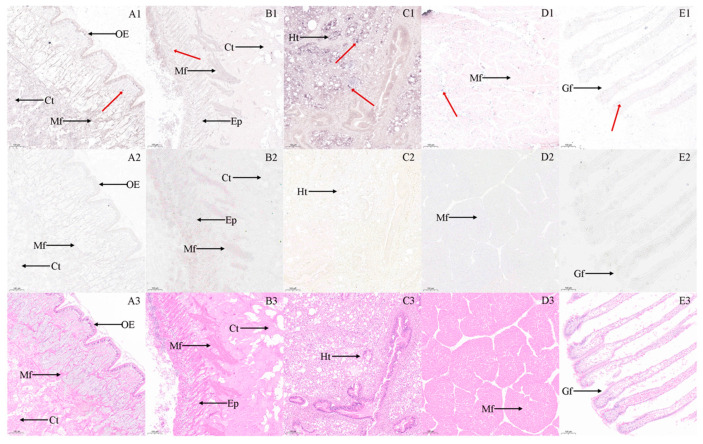
Cytological localization of *StAR* in foot, mantle, hepatopancreas muscle, and gill of ark shell. Note: Positive signals are indicated by the red arrow. (**A**): Foot; (**B**): mantle; (**C**): hepatopancreas; (**D**): muscle; (**E**): gill; (**1**): in situ hybridization results; (**2**): negative control diagram; (**3**): hematoxylin–eosin staining; OE: outer epidermis; Mf: muscle fiber; Ct: conjunctive tissue; Ep: epithelium; Ht: hepatic tubule; Gf: gill filament; bar: 100 µm.

In situ hybridization analyses of gonadal tissues (Figure 3) revealed distinct spatial expression patterns of the *StAR* gene. In the ovary, positive hybridization signals for the *StAR* gene were predominantly localized to the oocyte and follicle wall, with cytoplasmic hybridization signals in oocytes aligning well with predicted subcellular localization. In the testis, analyses demonstrated detectable *StAR* expression in both spermatogonia and spermatozoa. Extragonadal tissue localization results (Figure 4) indicated widespread *StAR* expression across multiple tissues. Compared with the negative control group, positive hybridization signals were primarily observed in the outer epidermis, muscle fibers, and conjunctive tissues of the foot (Figure 4A1,A2); in the mantle, hybridization signals were more pronounced in the muscle fibers, whereas weaker signals were detected in the epithelium and conjunctive tissue (Figure 4B1,B2); in the hepatopancreas, strong hybridization signals were evident on the hepatic tubules (Figure 4C1,C2); in the muscle tissue, signals were localized to the muscle fibers (Figure 4D1,D2); in the gill tissue, hybridization signals were detected on the surface of the gill filaments (Figure 4E1,E2). Comparative analysis with negative controls confirmed tissue-specific localization patterns. To validate the specificity of our hybridization assay, we included a no-probe control group (negative control: using hybridization buffer without probe), which showed a complete absence of signals. Tissue-specific expression analysis revealed markedly elevated *StAR* signal intensity in gonadal tissues compared to extragonadal tissues. Furthermore, probe specificity was rigorously verified through comprehensive BLAST analysis during the probe design phase, effectively excluding potential non-specific binding events.

### 3.3. Analysis of StAR Gene Expression in Gonads at Different Developmental Stages

**Figure 5:**
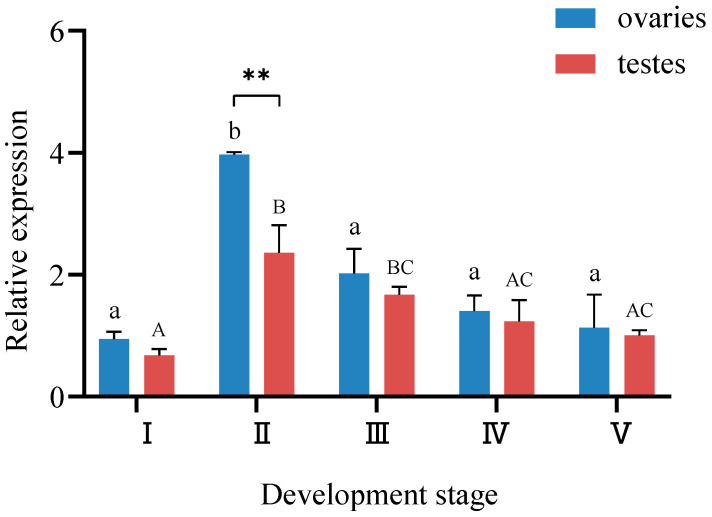
Relative expression of *StAR* in gonads during different developmental periods of ark shell. Note: ** indicates an extremely significant difference (*p* < 0.01). Different lowercase letters represent differences in ovarian expression across developmental periods, while different capital letters indicate differences in testicular expression across different periods.

The qRT-PCR analysis demonstrated ubiquitous *StAR* expression in both ovaries and testes across all five developmental stages (Figure 5). The highest ovarian *StAR* transcript levels were observed at stage Ⅱ, showing statistically significant elevation compared to other stages (*p* < 0.01). Similarly, testicular *StAR* expression peaked at stage Ⅱ, registering values significantly higher than those of stages Ⅰ, Ⅳ and Ⅴ (*p* < 0.01). Quantification of *StAR* expression dynamics revealed a progressive increased from stage Ⅰ to stage Ⅱ, peaking at Ⅱ, followed by a gradual decline through stage III to V, ultimately reaching minimal expression levels at stage V. Cross-sexual comparative analysis identified consistently elevated ovarian *StAR* expression relative to testes at matched developmental stages. The stage-specific sexual dimorphism exhibited maximal divergence at stage II (*p* < 0.01), while no statistically significant intersexual differences were observed at other developmental phases.

### 3.4. Steroid Hormone Profiles During Gonadal Development in Ark Shell

**Figure 6:**
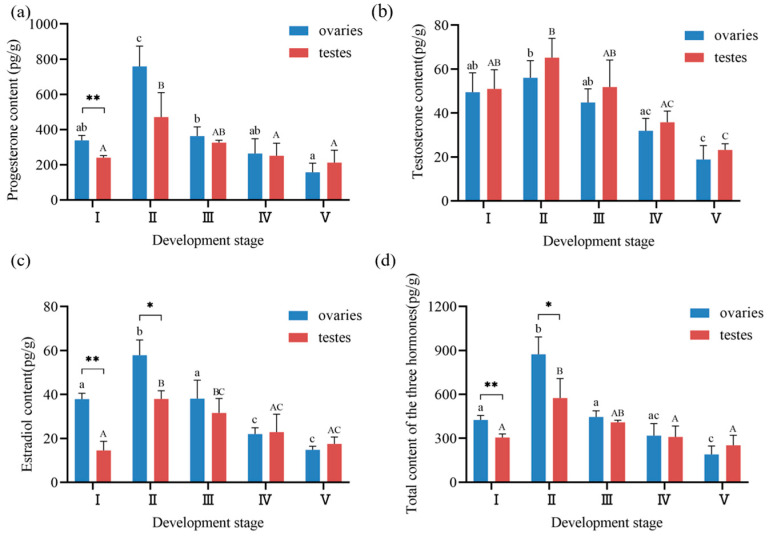
Sex steroid hormone content during gonadal development stages in ark shell. (**a**) Progesterone content across gonadal developmental stages. (**b**) Testosterone content across gonadal developmental stages. (**c**) Estradiol content across gonadal developmental stages. (**d**) Total content of the three hormones across gonadal developmental stages. Note: * indicates significant difference (*p* < 0.05) between testes and ovaries at the same stage; ** indicates a highly significant difference (*p* < 0.01). Different lowercase letters in the graphs represent the differences in ovarian hormone contents across stages, while different capital letters denote differences in testicular hormone contents across stages (applies to all panels).

As shown in Figure 6a, progesterone concentrations in ark shell gonads exhibited distinct variation patterns across five developmental stages. Ovarian progesterone content ranged from 157.04 ± 51.43 to 759.01 ± 114.99 pg/g, while testicular progesterone content varied between 211.81 ± 70.22 and 470.99 ± 138.94 pg/g. Both tissues showed peak concentrations at stage II, with ovarian levels significantly higher than those of other stages (*p* < 0.01), and testicular levels significantly elevated compared to stages I, IV, and V (*p* < 0.05). Progesterone content exhibited a pattern of increasing from stage I to stage II (reaching maximum values), then progressively declining through stages III-V (attaining minimum values at stage V). Notably, ovarian progesterone concentrations at stage Ⅰ were significantly higher than testicular levels at the same stage (*p* < 0.01). Although ovarian progesterone remained elevated compared to testes at most stages (excluding stage V), no significant intersexual difference was observed at stage II.

Figure 6b illustrates testosterone dynamics during gonadal development. Ovarian testosterone concentrations range from 18.86 ± 6.30 to 56.03 ± 7.84 pg/g, while testicular testosterone levels spanned 23.22 ± 2.78 to 65.17 ± 8.82 pg/g. Both sexes exhibited peak testosterone levels at stage II, significantly exceeding those of stage IV (*p* < 0.05) and stage V (*p* < 0.01). The temporal pattern mirrored progesterone dynamics, with concentrations peaking at stage II followed by progressive decline through stage V. A comparison of testosterone content differences between testes and ovaries across developmental stages revealed that testicular testosterone levels were consistently higher than ovarian levels at five developmental stages, albeit without reaching statistical significance.

As shown in Figure 6c, estradiol content in the ovaries ranged from 14.80 ± 1.66 to 57.90 ± 6.87 pg/g, and in testes from 17.52 ± 3.10 to 38.00 ± 3.70 pg/g. Ovarian estradiol content at stage Ⅱ was significantly higher than at other ovarian stages (*p* < 0.01), while testicular estradiol content at stage Ⅱ was significantly higher than that at the other four stages (*p* < 0.05). Estradiol concentrations followed a similar ascending–descending trajectory, peaking at stage II. Notably, ovarian estradiol at stages I and II demonstrated significant intersexual differences (*p* < 0.05), whereas testicular levels surpassed ovarian concentrations at stages III-V without significant difference.

The total gonadal hormone content is summarized in Figure 6d. In ovaries, total hormone content ranged from 190.70 ± 56.47 to 872.94 ± 117.97 pg/g, while in testes, it varied from 252.55 ± 68.12 to 574.16 ± 133.90 pg/g. Both tissues exhibited maximal total hormone concentrations at stage Ⅱ, with ovarian totals significantly higher than all other stages (*p* < 0.01), and testicular totals significantly elevated compared to stages I, IV, and V (*p* < 0.05). Total hormone content followed a characteristic pattern of peaking at stage II, followed by progressive decline. Significant intersexual differences were observed at stage I (*p* < 0.01) and stage II (*p* < 0.05), with ovarian totals consistently higher than testicular totals throughout development except at stage V.

### 3.5. Changes in Hormone Proportion with Gonadal Development

**Figure 7:**
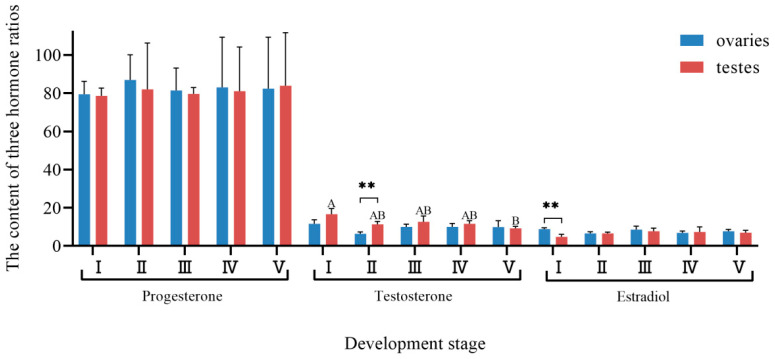
The content of the three hormone ratios in the gonad developmental periods of ark shell. Note: ** indicates a highly significant difference (*p* < 0.01) between testes and ovaries at the same stage. Different capital letters denote differences in testicular hormone contents across stages.

The percentage of progesterone, testosterone, and estradiol in the gonads of ark shell in the total hormone content was analyzed (Figure 7). Collectively, these three hormones accounted for approximately 80%, 10%, and 10% of the total hormone content, respectively. In ovaries, the proportion of progesterone peaked at stage Ⅱ and was minimal at stage Ⅰ. While ovarian progesterone percentages generally exceeded testicular values across all stages except stage Ⅴ, no significant interstage or intersexual differences were observed. In testes, the proportion of progesterone reached its maximum at stage Ⅴ and minimum at stage Ⅰ. No significant differences were detected in progesterone proportion between the five stages of testes and ovaries. For testosterone, the ovarian proportion was the highest at stage Ⅰ and lowest at stage Ⅱ. In testes, the proportion of testosterone peaked at stage Ⅰ and was minimal at stage Ⅴ. Except for stage Ⅴ, testicular testosterone proportions were higher than ovarian proportions, with significantly higher levels in testes compared to ovaries at stage Ⅱ (*p* < 0.01). Testicular testosterone proportions at stage Ⅰ were significantly higher than those at stage Ⅴ (*p* < 0.05), while no significant difference was observed in testosterone levels among different ovarian developmental stages. Estradiol proportions in ovaries were highest at stage Ⅰ and lowest at stage Ⅳ. Ovarian estradiol proportions were slightly higher than testicular proportions except in stage Ⅳ, with significantly higher levels in ovaries compared to testes at stage Ⅰ (*p* < 0.01). In testes, estradiol proportions peaked at stage Ⅲ and were minimal at stage Ⅰ. No significant differences were detected in estradiol proportions between testes and ovaries across the five stages.

### 3.6. Correlation Among Hormones and Between Hormone and Gene Expression Levels

**Figure 8:**
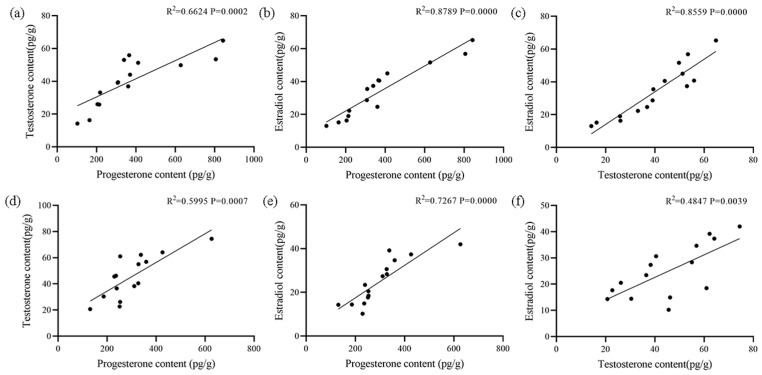
Correlation analysis of three sex steroid hormones in the ovaries and testes of ark shell. (**a**) Correlation between progesterone content and testosterone content in ovaries. (**b**) Correlation between progesterone content and estradiol content in ovaries. (**c**) Correlation between testosterone content and estradiol content in ovaries. (**d**) Correlation between progesterone content and testosterone content in testes. (**e**) Correlation between progesterone content and estradiol content in testes. (**f**) Correlation between testosterone content and estradiol content in testes.

**Figure 9:**
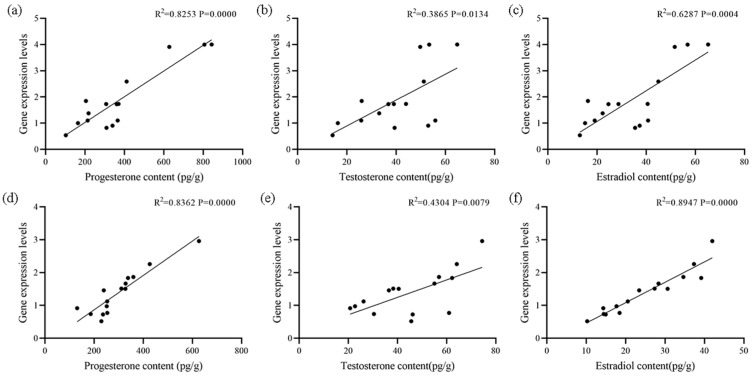
Correlation analysis of sex steroid hormones and *StAR* gene expression levels in the ovaries and testes of ark shell. (**a**) Correlation between progesterone content and *StAR* gene expression levels in ovaries. (**b**) Correlation between testosterone content and *StAR* gene expression levels in ovaries. (**c**) Correlation between estradiol content and *StA*R gene expression levels in ovaries. (**d**) Correlation between progesterone content and *StAR* gene expression levels in testes. (**e**) Correlation between testosterone content and *StAR* gene expression levels in testes. (**f**) Correlation between estradiol content and *StAR* gene expression levels in testes.

The correlation results of the three sex steroid hormones in ovaries and testes are shown in Figure 8. In ovaries, there was a strong and significant positive correlation among the three sex steroid hormones (*p* < 0.01), with correlation coefficients of 0.6624, 0.8789, and 0.8559, respectively. In testes, the contents of progesterone and testosterone or estradiol were significantly positively correlated (*p* < 0.01), while contents of testosterone and estradiol were moderately positively correlated (*p* < 0.05). The correlation results between the contents of the three sex steroid hormones and the expression level of the *StAR* gene in ovaries and testes, respectively, are shown in Figure 9. In ovaries, there was an extremely significant strong positive correlation (*p* < 0.01) between progesterone and estradiol contents and the *StAR* gene expression level, as well as a significant moderate positive correlation (*p* < 0.05) between the testosterone content and the *StA*R gene expression level. In testes, progesterone and estradiol content showed an extremely significant strong positive correlation (*p* < 0.01) with the *StAR* gene expression level, whereas testosterone contents exhibited a significant moderately positive correlation (*p* < 0.01) with the *StAR* gene expression level. In both ovaries and testes, there was an extremely significant strong positive correlation between the *StAR* gene expression level and the contents of progesterone and estradiol (*p* < 0.01), suggesting that the *StAR* gene may positively regulate the levels of these hormones.

## 4. Discussion

Sex steroid hormones are ubiquitously present in Mollusks and crustaceans, and their concentrations fluctuate across different reproductive stages, exhibiting sexual dimorphism. These hormones play a critical role in sex determination and gonad development [24,25,26]. The *StAR* mediates the translocation of cholesterol from the outer mitochondrial membrane to the inner mitochondrial membrane, which represents the first and one of the rate-limiting steps in sex steroid hormone synthesis [27]. Initially cloned from the mouse MA-10 Leydig cell line, the *StAR* protein exhibits a highly conserved structure characterized by hydrophobic amino acids at the N-terminal region and a conserved START domain at the C-terminal end [28]. In this study, the CDS of the *StAR* gene isolated from ark shell was 1446 bp in length, encoding a protein of 481 amino acids. Furthermore, the *StAR* protein was found to possess similar N-terminal, C-terminal, and *STAR*T conserved domains, suggesting that the *StAR* protein is structurally and functionally conserved and plays a pivotal role in transport across diverse organisms. The *StAR* is an essential regulatory factor in the steroid hormone synthesis process of vertebrates, playing a regulatory role in the growth, differentiation, development, and reproduction, particularly in promoting the development of reproductive organs and maintaining spermatogenic function [29]. In situ hybridization results showed that in the ovary of ark shell, the positive hybridization signals of the *StAR* gene were mainly concentrated in the oocytes and follicular walls. The cytoplasmic hybridization signals in oocytes were highly consistent with the predicted subcellular localization. However, in mammalian ovaries, *StAR* is mainly expressed in theca cells, which are responsible for transporting cholesterol to mitochondria for androgen synthesis, while granulosa cells further convert androgens into estrogens [30]. This may reflect the evolutionary divergence in steroid hormone synthesis pathways between invertebrates and vertebrates. In mammals, existing studies have confirmed that the expression of the *StAR* gene in theca cells increases with the elevation of follicle maturity, and this expression pattern is synchronized with the peak of steroid hormone synthesis [31]. During the gonadal development of bivalves, the expression patterns of steroidogenesis-related genes are also accompanied by spatiotemporal-specific changes in cell types. Studies have found that in bivalves, the activity and expression location of steroidogenic enzymes vary across different stages of gonadal development [13]. Based on the results of this study, we hypothesize that the types of cells expressing *StAR* in ark shell may dynamically adjust throughout the process of gonadal development. In this study, the expression of *StAR* in stage Ⅱ ovaries and testes was the highest, and its expression varied with the gonadal development of ark shell. During the transition from the later active stage to mature stages in the ovaries and testes of ark shell, the expression of *StAR* decreased rapidly, suggesting that the *StAR* gene may play an important role in the maturation and discharge of germ cells. The *StAR* gene exhibited relatively higher expression during the early stage of gonadal differentiation, indicating its involvement in the early gonadal development of *H. cumingii* [32]. A high expression of the *StAR* gene was observed in the testes of *Macrobrachium nipponense*, and its expression was significantly upregulated under hypoxia, suggesting that *StAR* may influence gamete maturation in crustacean reproductive regulation via the steroid hormone pathway [33]. Testosterone and 17β-estradiol were present in the hemolymph of the Barents Sea red king crab (*Paralithodes camtschaticus*). It has been found that testosterone levels exhibit seasonal variations, peaking during the spawning period, suggesting that testosterone may play an important role in the reproductive cycle of the red king crab. In contrast, the function of 17β-estradiol may be more closely related to ovarian activities [26]. In the gonads of ark shell, the expression of the *StAR* gene reached its peak at stage Ⅱ, consistent with the highest levels of progesterone, testosterone, estradiol, and total content. Moreover, the trend of *StAR* expression closely mirrored the trend of the contents and total contents of three sex steroid hormones during gonadal development. These findings indicate that as a sex steroid hormone synthesis gene, *StAR* may influence the gonadal development of ark shell by modulating the synthesis of sex steroid hormones, and that sex steroid hormones and synthesis-related genes may play a significant role in germ cell development.

Sex steroid hormones play pivotal roles in regulating reproductive cycles in vertebrates [34], yet their seasonal dynamics and functional significance in shellfish remain poorly characterized. In this study, ELISA was employed to detect the contents of three sex steroid hormones in the gonads of ark shell, and the results indicated that the contents of sex steroid hormones varied seasonally with gonadal development. From stage Ⅰ to stage Ⅱ, the three hormone contents increased, while from stage Ⅱ to stage Ⅴ, they decreased. Additionally, the content of sex steroid hormones in the gonads of other shellfish also exhibited seasonal variation with gonad development. The contents of estradiol and testosterone in *C. farreri* varied periodically with the reproductive process, suggesting that the sex steroid hormone plays a potentially important role in sex maintenance, gonadal differentiation, gametogenesis, and release [35]. The contents of testosterone and estradiol in the gonads of *C. ariakensis* were the highest during the early active stage, and changes in sex steroid hormones were related to the reproductive cycle, suggesting that testosterone and estradiol may be involved in regulating gonadal development [31]. In this study, the content of sex steroid hormones in the gonads of ark shell varied seasonally with the reproductive cycle, demonstrating the importance of sex steroid hormones in regulating reproduction.

Sex steroid hormones are widely distributed in shellfish, and their concentrations exhibit sexual dimorphism, playing crucial roles in sex determination and gonad development [3]. Some studies have shown that sex steroid hormones can accelerate gonadal development and influence the sex of shellfish [36]. The results of this study revealed that the content of three sex steroid hormones in the gonads differed between ovaries and testes, with significant differences observed during certain periods, showing sexual dimorphism. In *M. yessoensis* ovaries, the contents of estradiol were higher than in testes, whereas the content of testosterone in testes was higher than in ovaries [7]. During gonadal development in *A. irradians*, progesterone and estradiol contents in ovaries were higher than those in testes, and testosterone contents in testes were significantly higher than those in ovaries, showing sexual dimorphism [37]. In several shellfish species, the contents of estrogens (testosterone and estradiol) in ovaries and androgens (testosterone) in testes were higher, and the sex dimorphism of these hormone contents suggested that the functions of estrogens and androgens in ovaries and testes may differ by sex. The results showed that the contents of these three hormones varied among the gonads of ark shell, *M. yessoensis*, *C. farreri*, *A. irradians*, *C. gigas,* and *C. ariakensis*. The hormone contents in the gonads of *M. yessoensis* and *A. irradians* were higher than those in *C. farreri*, *C. gigas,* and *C. ariakensis*, while the hormone contents in the gonads of ark shell were significantly lower than those in the three scallops, but only slightly different compared with those in the two oysters. These differences may result from variation in species specificity, hormone extraction and detection methods, and stages of gonad development. In summary, sex steroid hormones exhibit distinct sexual dimorphism across shellfish species, underscoring their critical roles in sex-specific reproductive functions.

The expression of the *StAR* gene is closely associated with the synthesis of sex steroid hormones, as it promotes steroid hormone production by regulating the transport of cholesterol into mitochondria [38]. This study revealed strong positive correlations among *StAR* and sex steroid hormones in gonads, with particularly significant associations between progesterone/estradiol and *StAR* gene expression in both ovaries and testes. *FOXL2* expression positively correlates with estradiol levels and the estradiol/testosterone ratio in *A. irradians*, while *DMRT1L* expression positively correlates with testosterone but negatively with estradiol/testosterone, suggesting their potential roles in regulating gonadal steroid hormones to influence gonadal development and sex differentiation [8]. In Nile tilapia, estradiol and testosterone injections significantly elevated serum estradiol and testosterone levels, with estradiol enhancing female growth performance and upregulating *ghr1*, *ghr2*, *igf1*, and *igf2*, while testosterone promoted male growth and increased these genes plus the *muscle regulatory factor*, indicating a close relationship between sex steroid hormones and related gene expression [39]. This study demonstrated that both progesterone and estradiol levels exhibit highly significant strong positive correlation with *StAR* gene expression, while testosterone shows a significant moderate positive correlation, indicating that *StAR* plays crucial regulatory roles in sex steroid hormone synthesis.

## 5. Conclusions

This study characterized the tissue-specific distribution and gonadal expression profiles of the *StAR* gene in the ark shell, while simultaneously quantifying three principal sex steroid hormones (progesterone, testosterone, and estradiol) throughout gonadal development. The results demonstrated that *StAR* potentially regulates gonadal maturation by modulating steroid hormone biosynthesis. Notably, progesterone and estradiol levels exhibited strongly positive correlations with *StAR* expression, confirming *StAR*’s fundamental regulatory role in steroidogenesis. These findings provide novel insights into the endocrine mechanisms governing bivalve reproduction, which has implications for aquaculture management and conservation.

## Figures and Tables

**Table 1:** Primer sequences required for the experiment.

Prime	Sequence (5′-3′)	Function
StAR-F	CGTGAGAGATGTTGCAAGCATTCA	validation
StAR-R	ATCACAATGTTCCATCCAATGGCA	validation
q-StAR-F	TTTAACGCCAGTAAAGCAGGAGGAG	qRT-PCR
q-StAR-R	CAAGTCTCTACCCACGCCAACAC	qRT-PCR
RL15-F	AGACCAGACAAAGCCAGAAGAC	qRT-PCR
RL15-R	GCTGAAGTAAGTCCACGCATT	qRT-PCR
StAR	UCUAAGAACCAAGUCUCUACCCACGCCAA	in situ hybridization

**Table 2:** *StAR* amino acid sequence numbers and identity of different species.

Taxonomic Status	Species Name	Sequence Number	Homology
Shellfish	*Mizuhopecten yessoensis*	XP_021369546.1	51.45%
	*Mimachlamys nobilis*	AJM13632.1	51.24%
	*Pecten maximus*	XP_033733178.1	50.21%
	*Hyriopsis cumingii*	WEY07736.1	49.78%
	*Mercenaria mercenaria*	XP_053408879.1	46.17%
	*Dreissena polymorpha*	XP_052253900.1	51.85%
	*Mya arenaria*	XP_052804046.1	51.38%
	*Ostrea edulis*	XP_048727697.1	48.77%
	*Crassostrea angulata*	XP_052718357.1	47.51%
	*Crassostrea gigas*	XP_034305144.1	47.51%
Fish	*Salmo salar*	ABD73012.1	33.19%
	*Danio rerio*	AAG28593.1	35.68%
	*Cynoglossus semilaevis*	AIB06798.1	33.33%
Amphibian	*Xenopus laevis*	NP_001167502.1	35.15%
	*Andrias davidianus*	AUS91513.1	32.70%
	*Pelophylax nigromaculatus*	AVP72471.1	38.61%
Bird	*Gallus gallus*	AAG28594.1	33.01%
	*Taeniopygia guttata*	AAR91038.1	32.31%
	*Rissa tridactyla*	XP_054081021.1	32.86%
Mammal	*Bos taurus*	CAA76718.1	33.65%
	*Mus musculus*	AAB94783.1	33.18%
	*Homo sapiens*	AAB88174.1	34.12%

## Data Availability

The original contributions presented in this study are included in the article. Further inquiries can be directed to the corresponding author.
